# Simultaneous Adsorption of Cu^2+^ and Cd^2+^ by a Simple Synthesis of Environmentally Friendly Bamboo Pulp Aerogels: Adsorption Properties and Mechanisms

**DOI:** 10.3390/polym14224909

**Published:** 2022-11-14

**Authors:** Wenxiang Jing, Lijiang Yin, Xiaoyan Lin, Ying Yu, Dongming Lian, Zhaoming Shi, Peng Chen, Min Tang, Chai Yang

**Affiliations:** 1 Engineering Research Center of Biomass Materials, Ministry of Education, Southwest University of Science and Technology, Mianyang 621002, China; 2 Yibin Industrial Academy of Forestry and Bamboo, Yibin 644005, China

**Keywords:** bamboo-based pulp aerogel, tannin, chitosan, simultaneous adsorption, heavy metal ions

## Abstract

The highly efficient, pollution-free and degradable biomass-based adsorbents used for the purification of wastewater are currently being highlighted in the research. Bamboo is an excellent raw material for pulp production due to its characteristics of fast growth, wide distribution and high cellulose content. In this study, a tannin/chitosan/bamboo pulp aerogel (TCPA), an environmentally friendly, renewable and low-density adsorbent, was synthesized using a simple freeze-drying method and analyzed by FTIR, XPS, SEM, TEM, TGA and surface area and porosity methods. TCPA has a large specific surface area (137.33 m^2^/g) and 3D porous structure, and its surface has multiple functional groups including amino, carboxyl and hydroxyl groups, which lead to a simultaneous absorption effect with Me^2+^ (Cu^2+^ and Cd^2+^). The maximum adsorption capacity for Cu^2+^ and Cd^2+^ of the TCPA was 72.73 mg/g and 52.52 mg/g, respectively. The adsorption processes of Me^2+^ by TCPA follow the pseudo-second-order model and Langmuir isotherm mode, and the adsorption processes are spontaneous and endothermic. The study provides a promising candidate for the treatment of wastewater containing heavy metal mixtures.

## 1. Introduction

With rapid industrialization, a large amount of wastewater containing heavy metals is being discharged into the environment, causing water pollution over a large area and harming the ecological environment [1]. Therefore, it is imperative to remove heavy metals from all kinds of polluted water by using efficient, non-polluting and easily degradable adsorbents. There are three ways to remove heavy metal pollution from water bodies; the first is the chemical treatment method [2,3,4], but it can easily cause problems such as secondary pollution, and limited types of heavy metals that can be treated; the second is using a biological treatment method [5,6,7], which has excellent adsorption properties, but has long treatment cycles and high costs; the third is an adsorption method [8,9,10], in which heavy metal ions in a polluted system are adsorbed whilst maintaining the original chemical morphology of the heavy metals.

In the bamboo industry, bamboo pulp is mainly produced using a pulping process [11,12]. It requires high cellulose content and low content of other components, such as hemicellulose, lignin, extract, etc., as well as high levels of whiteness and relatively uniform molecular mass distribution [13]. After undergoing a bleaching treatment, the cellulose content of the prepared pulp can reach over 90%, the hemicellulose content can be reduced to less than 6%, the lignin content is less than 2% and the ash content to less than 1% [14,15]. It is mainly used to produce synthetic fibers including viscose fibers [16], lyocell fibers [17], acetic acid fibers [18], copper–ammonia fibers [19], etc. Due to the low solubility of cellulose in common solvents, only microcrystalline cellulose fibers [20] or nano-cellulose fibers [21] are usually used as raw materials for the preparation of aerogels at present. In addition, a variety of aerogels have been reported, such as graphene aerogel [22], silica aerogel [23], carbon aerogel [24], etc., but most of them have certain disadvantages, such as high price, poor regeneration, poor environmental friendliness and so on.

Chitosan is a natural biopolymer derived from crustaceans, fungi, etc. It is the only natural alkaline polysaccharide that has been discovered to date. Chitosan and its modified molecules contain a large number of hydroxyl and amino groups, which play a strong role in chelating metal ions; therefore, it can be used to recover and treat heavy metal ions from industrial wastewater and is considered a “green water treatment agent “ [25]. Tannin, a plant polyphenol, is a complex phenolic secondary metabolite in the plant body, has a polyphenolic structure and has a strong ability to form complexes with metal ions [26,27,28]. Polyphenols are mainly found in the bark, root, leaf, shell and sarcocarp of plants. They are abundant in nature. At present, the annual tannin production in the world is about 250,000 to 300,000 tons [29]. The literature shows that the immobilization of tannin on media such as cellulose, aminopolystyrene and agar can be used as an adsorbent material with a high adsorption capacity for many metal ions [30]. Hu et al. [31] proposed a new type of adsorbent made of carbon disulfide-modified tannic acid gel for the adsorption of Cu(Ⅱ) ions. Similarly, amino groups can adsorb heavy metals.

In this paper, the bamboo pulp was used as the base material, and monomers of tannin and chitosan, both of which have a chelating effect on metal ions, were mixed simply. Then, tannin/chitosan/bamboo pulp aerogel adsorbents (TCPA) were prepared using a freeze-drying method. The materials were analyzed by FTIR, XPS, SEM, TEM, TGA and a surface area and porosity analyzer. Cu^2+^ and Cd^2+^ were used as adsorbents, and the effects of additive type, pH, initial concentration and adsorption temperature on the adsorption performance of Cu^2+^ and Cd^2+^ were explored. The adsorption process of TCPA for Cu^2+^ (or Cd^2+^) was accurately described by the adsorption kinetics; adsorption thermodynamics described the relationship between Cu^2+^ (or Cd^2+^) in TCPA and in solution. Therefore, the results showed that TCPA exhibited the characteristics of low price and environmental friendliness with a high adsorption capacity for Cu^2+^ and Cd^2+^.

## 2. Materials and Methods

### 2.1. Chemicals and Materials

Bamboo pulp was obtained from Sichuan Tianzhu Bamboo Resources Development Co., Ltd., Yibin, China. Tannin (Gallotannin, Mw = 1701.2 g/mol) and chitosan (medium molecular weight) were purchased from Sigma-Aldrich L.L.C., Shanghai, China. N-methyl morpholine-N-oxide, NMMO (AR, purity ≥ 99%) was purchased from Bide Pharmatech Ltd., Shanghai, China. Cu(NO_3_)_2_ (AR, purity ≥ 99%) and Cd(NO_3_)_2_ (AR, purity ≥ 99%) were purchased from West Asia Chemical Technology (Shandong) Co., Ltd., Linyi, China. Ultra-pure water was self-made, with resistivity ≥ 18.25 MΩ·cm.

### 2.2. Preparation of Bamboo Pulp Aerogel

Bamboo pulp (0.5 g), additive (0.04 g), water (3.04 mL) and NMMO (20 g) were mixed in a beaker. The mixture was heated for 120 min at 90 °C. When the mixture was completely dissolved, the stirrer was turned on, and a certain amount of tannin or chitosan was slowly added. The pre-gel was stored at room temperature overnight. Then NMMO in the pulp pre-gel was replaced by soaking in deionized water. Finally, the hydrogel was pre-frozen at −24 °C and freeze-dried (−60 °C) for 48 h. The reagent dosage in the fabrication procedure is listed in Table 1.

### 2.3. Characterization

The morphology analysis of the samples was carried out by SEM (Regulus 8100, Hitachi, Tokyo, Japan) and TEM (Tecnai G2 F20, FEI, Hillsboro, USA). The specific surface area/porosity of the samples was determined by the BET surface area measurement method (ASAP 2020, Micromeritics, Norcross, USA). The DFT model was used to analyze the porosity. The surface functional groups and composition were analyzed by XPS (Escalab 250Xi, ThermoFisher, Waltham, USA) and FTIR (IRAffinity-1S, Shimadzu, Kyoto, Japan). The thermal stability of samples was tested by TG-DSC (SAT 449C, Netzsch, Germany) at the heating rate of 10 °C/min in nitrogen.

### 2.4. Adsorption Experiment with Cu^2+^ and Cd^2+^

Cu^2+^ and Cd^2+^ in an aqueous solution were used as adsorbates to evaluate the adsorption performance of the samples. The aqueous solution was adjusted to the desired pH by 0.1 mol/L sodium hydroxide or hydrochloric acid. Then 0.05 g of sample was put into a conical bottle, and 20 mL of the solution with 5–15 μg/mL of Cu^2+^ (or Cd^2+^) was added. The conical bottle was placed in the shaker (WHY-2A, KEXI Instrument, Changzhou, China) to adsorb with shaking at 120 rpm/min for 420 min. The concentrations of Cu^2+^ and Cd^2+^ were determined by Atomic Absorption Spectrophotometry (PinAAcle 900T, PerkinElmer, Waltham, MA, USA) at regular intervals. The adsorption capacity (*q_t_*) was calculated using Equation (1) and the removal percentage (*η*) was calculated using Equation (2). The experiments were replicated three times.

(1) $$q_{t}(\mathrm{mg}/g)=\frac{(C_{0}-C_{1})\times V}{m}$$

(2) $$\eta \left(\%\right)=\frac{C_{0}-C_{1}}{C_{0}}\times 100$$

where *C*_0_ is the concentration of Cu^2+^ or Cd^2+^ in the solution before adsorption, mg/mL; *C*_1_ is the concentration of Cu^2+^ or Cd^2+^ in the solution after adsorption, mg/mL; *V* is the volume of sample solutions, mL; *m* is the weight of samples, g.

### 2.5. Regeneration of Aerogels

After the adsorption experiment, the TCPAs were removed from the solution. First, the surface adhered free states of Cu^2+^ or Cd^2+^ were removed by immersion in deionized water for 2 h. Then, the samples were eluted in an aqueous 0.1 mol/L HCl solution and freeze-dried. These processes were repeated three times, and the corresponding removal percentage was obtained each time.

## 3. Results and Discussion

### 3.1. Characterization

**Figure 1:**
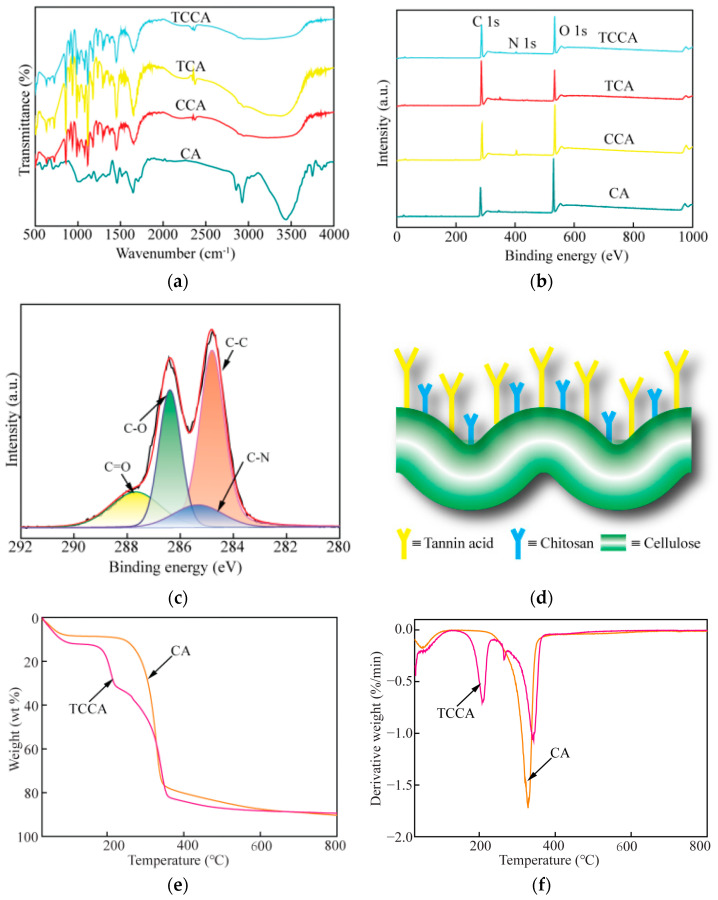

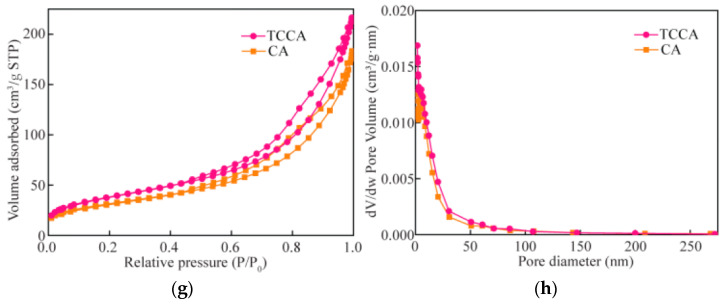
(**a**) FTIR spectra and (**b**) wide scan XPS spectra of PA, TPA, CPA and TCPA; (**c**) C1s of TCPA; (**d**) schematic diagram of TCPA, (**e**) TGA and (**f**) DTG of PA and TCPA; (**g**) N_2_ adsorption/desorption isotherm of PA and TCPA; (**h**) Pore size distribution of PA and TCPA.

The functional groups and elemental composition on the surface of samples PA, CPA, TPA and TCPA were determined by FTIR and XPS. The results are shown in Figure 1a–c. The stretching vibration of -OH, -NH- and -NH_2_ was observed at 3450 cm^−1^. The band located around 3600–2800 cm^−1^ became broader, which indicated that hydrogen bonding was enhanced, both with tannin and chitosan. The stretching vibration of C-H and -CH_2_ were observed at 2854 cm^−1^ and 2935 cm^−1^, respectively. The peak at 1655 cm^−1^ represented the -C=O stretch vibration and the vibration of the -NH_2_ bend. The peak at 1118 cm^−1^ represented the stretching vibration of C-O-C [32,33,34]. Figure 1b shows the different samples that were analyzed by XPS. Compared with PA and TPA, N1s peaks determined by the wide scan XPS spectra could be clearly found in TCPA and CPA due to the addition of chitosan. In addition, the C/O ratio in TPA than PA was obviously elevated, which indicated that tannin was successfully combined with bamboo pulp. The results are presented in Table 2. Figure 1c shows the C1s spectrum of TCPA with four peaks displayed representing C-C (284.8 eV), C-N (285.3 eV), C-O (286.4 eV) and C=O (287.7 eV). In conclusion, the hydroxyl groups on the surface of the aerogels were successfully combined with chitosan and tannin on the surface of aerogels by hydrogen bonding which is consistent with the reported literature [35,36,37]. A schematic diagram of TCPA is shown in Figure 1d.

Figure 1e,f shows the TGA and DTG curves of PA and TCPA. Figure 1e shows that the weight loss curve of PA and TCPA was divided into two main stages. In the first stage, the weight loss of the sample that occurred from room temperature to 100 °C was due to the water in the sample being evaporated [38]. The weight loss of TCPA and PA was 12% and 8%, respectively. The second stage of weight loss mainly occurred at around 200~400 °C, and the weight loss of TCPA and PA was 76% and 82%, respectively. Compared with PA, TCPA had a large weight loss at 163–224 °C, which might have been due to the formation of new substances from tannin and chitosan with the aerogels. The rate of weight loss was the largest at 208 °C, as shown in Figure 1f. The total weight loss of TCPA and PA was 88% and 90%, respectively, and the maximum weight loss rate was reached at 341 °C and 328 °C, respectively. This may have been due to the reaction between the pulp and the additives (tannin and chitosan), which resulted in polymers of a higher molecular weight. Thus, the thermal stability of the obtained composites was improved.

Figure 1g,h and Table 3 show the specific surface area and porosity of TCPA and PA. Figure 1g shows the N_2_ adsorption/desorption curves of TCPA and PA. In the first section of the adsorption curve (low pressure), the nitrogen adsorption capacity increased slowly with the increase of relative pressure. For the second section (higher pressure), the N_2_ adsorption capacity increased rapidly with the increase of relative pressure. According to IUPAC classification specifications, the above curves of PA and TCPA belong to type II with H2 hysteresis loops, indicating the presence of mesopores in both TCPA and PA and the phenomenon of capillary condensation and multilayer adsorption [39]. Figure 1h shows the pore size distribution of TCPA and PA. These data were obtained on the basis of the NLDFT. The pore sizes of PA and TCPA were mainly distributed in the range of 1.7–100 nm, which indicated that most of the pores were micro- and medium pores, and there were only a few large pores. In addition, as shown in Table 3, with the addition of tannin and chitosan, the specific surface area of TCPA increased by 21.96% compared with that of PA, and the total pore volume increased by 19.77%, within which the micropore and mesopore volumes (V < 50 nm) increased by 29.21%. The synergic effect of the microporous structure and the active sites played an important role in the adsorption process of heavy metals. Regarding the pore diameter (Dpore) and density (ρ), there was little difference between the TCPA and PA. These data indicated that the modification of chitosan and tannin enriched the pore structure of the bamboo pulp aerogels, which was consistent with the results of SEM.

**Figure 2:**
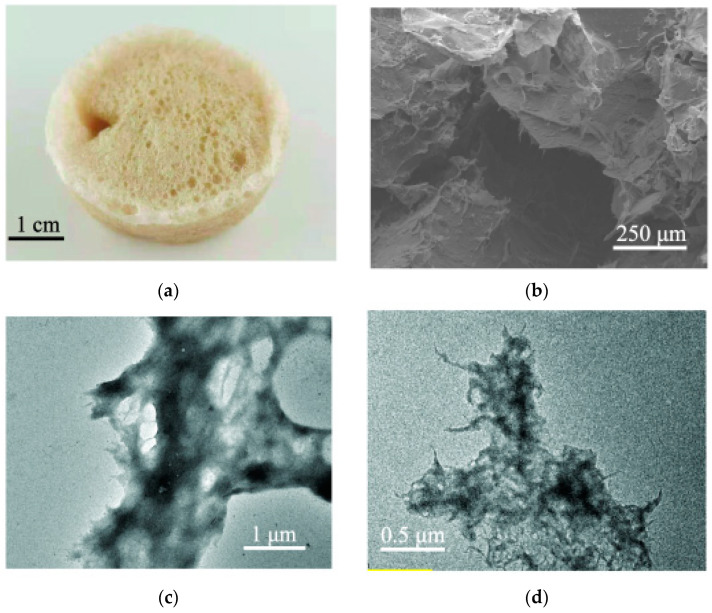

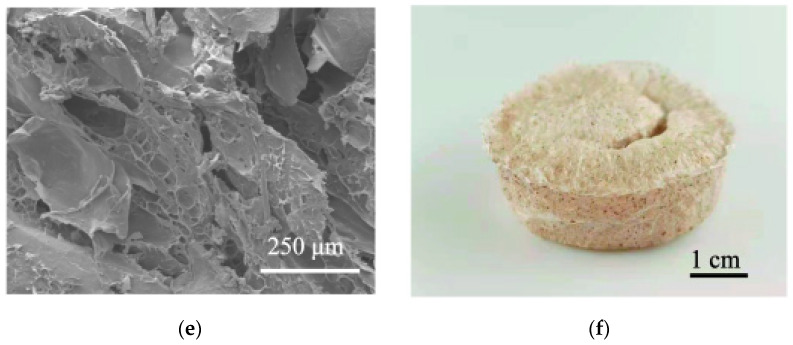
The digital images of (**a**) PA and (**f**) TCPA; SEM of (**b**) PA and (**e**) TCPA; TEM of (**c**) PA and (**d**) TCPA.

The surface ultrastructures of PA and TCPA were analyzed by SEM and TEM, as shown in Figure 2. Digital images (Figure 2a,f) showed that TCPA was darker colored than PA, which may be due to the formation of colored substances caused by heating tannins. The SEM spectra of PA and TCPA (Figure 2b,e) showed that both of them contained 3D pores with evenly distributed pores [37]. However, compared with PA, TCPA had more honeycomb structures, which indicated that more pores were formed by the addition of tannin and chitosan. The TEM spectrums of PA and TCPA showed that the samples were homogeneous and the additives did not separate and reunite, indicating that the process of dissolving was successful.

### 3.2. Adsorption Studies of Cu^2+^ and Cd^2+^

#### 3.2.1. Effect of Additive Types on Adsorption of Cu^2+^ or Cd^2+^ Solution

**Figure 3:**
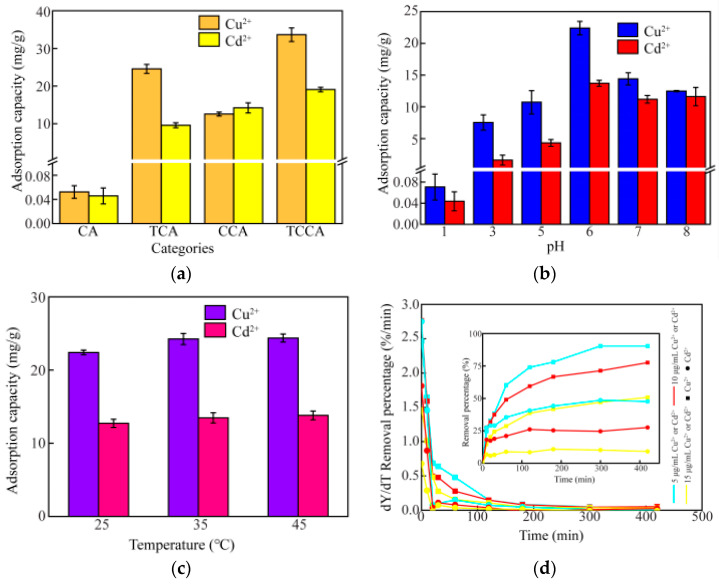
(**a**) Adsorption capacity of Cu^2+^ or Cd^2+^ by the TAPC at a concentration of 10 μg/mL, pH = 6.0, adsorption time = 420 min, adsorption temperature = 25 °C; (**b**) effect of pH values on adsorption capacity of Cu^2+^ or Cd^2+^ by the TCPA at the concentration of 5 μg/mL, adsorption time = 420 min, adsorption temperature = 25 °C; (**c**) effect of temperature on adsorption capacity of Cu^2+^ or Cd^2+^ by the TCPA at the concentration of 5 μg/mL, pH = 6.0, adsorption time = 420 min; (**d**) removal rate and removal percentage (insert) of Cu^2+^ or Cd^2+^ by the TCPA at pH = 6.0, adsorption time = 420 min, adsorption temperature 25 °C.

A series of bamboo pulp aerogels were successfully synthesized, and the adsorption performance was evaluated with wastewater containing Cu^2+^ or Cd^2+^. As shown in Figure 3a, the adsorption capacity of PA for Cu^2+^ or Cd^2+^ was very low. When tannin or chitosan was added to the bamboo pulp aerogels, the absorption capacity of Cu^2+^ or Cd^2+^ was significantly increased. An interesting phenomenon was observed, TPA had significantly more adsorption capacity of Cu^2+^ than CPA. On the contrary, CPA had significantly more adsorption capacity on Cd^2+^ than TPA. In addition, the absorption capacity of Cu^2+^ and Cd^2+^ by TCPA increased compared with the previously modified aerogels. This may have been due to the blending of the tannin, chitosan and pulp providing more binding sites [39].

#### 3.2.2. Effect of pH on Adsorption of Cu^2+^ or Cd^2+^ Solution

Figure 3b shows that the pH of the solution had a significant effect on the adsorption energy of Cu^2+^ and Cd^2+^. As the pH values increased, the rule of rising first and then decreasing was observed. Under the condition of low pH, the absorption capacity of TCPA to Cu^2+^ and Cd^2+^ was lower. The protonation of functional groups on the adsorbents was the main reason for the rejection of Cu^2+^ and Cd^2+^, thus leading to low adsorption capacities. As the pH value increased in the solution, a rapid rise was observed for Cu^2+^ and Cd^2+^ adsorption capacity, and the negative charge on the surface of the agent increased, so Cu^2+^ or Cd^2+^ could be captured by the adsorbents [40]. When pH > 6.0, Cu^2+^ and Cd^2+^ exist as hydrolytic substances, so the adsorbent’s adsorptive capacity decreased. Therefore, when the optimum pH value was 6.0, the adsorption capacities for Cu^2+^ and Cd^2+^ were the highest, 22.41 mg/g and 13.71 mg/g. The results showed that TCPA had a high synergistic adsorption capacity for Cu^2+^ and Cd^2+^.

#### 3.2.3. Effect of Temperature on Adsorption of Cu^2+^ or Cd^2+^ Solution

Figure 3c shows that the adsorption capacity of TCPA for Cu^2+^ or Cd^2+^ increased with increasing adsorption temperature, indicating that the increase in temperature promoted the adsorption process of TCPA for Cu^2+^ or Cd^2+^. In order to better understand the energy changes during the adsorption process, the thermodynamic parameters of the adsorption process were calculated by Equations (3)–(6).

(3) $$lnK=\frac{\Delta S^{\theta }}{R}-\frac{\Delta H^{\theta }}{RT}$$

(4) $$\Delta G^{\theta }=-RTlnK$$

(5) $$\Delta G^{\theta }=\Delta H^{\theta }-T\Delta S^{\theta }$$

(6) $$K=\frac{q_{e}}{C_{e}}$$

where *R* is the ideal gas constant, 8.314 J/(mol·K); *T* is the absolute temperature, K; Δ*G^θ^* is the change in Gibbs free energy, J/K; Δ*H^θ^* is the change in enthalpy, J/K; Δ*S^θ^* is the change in entropy, J/(mol·K); *K* is the equilibrium constant, (L/mol); *q_e_* is the amount of Cu^2+^ or Cd^2+^ in the TAAC at equilibrium; *C_e_* is the concentration of Cu^2+^ or Cd^2+^ in the solution at equilibrium, mg/L.

Table 4 shows that the Δ*G^θ^* of Cu^2+^ or Cd^2+^ by TCPA were negative, indicating a spontaneous adsorption process for Cu^2+^ or Cd^2+^ on TCPA. Δ*H^θ^* > 0, which reflected the endothermic of the adsorption process. Δ*S^θ^* > 0 represented the increase in the degree of freedom of the solid–solution interface during the adsorption process [41].

#### 3.2.4. Effect of the Concentration on Adsorption of Cu^2+^ or Cd^2+^

The initial concentration of Cu^2+^ (or Cd^2+^) was one of the key parameters that affected the performance of the adsorbent. Therefore, TCPA was used to test the removal rate and removal percentage (insert) of Cu^2+^ or Cd^2+^ with different initial concentrations (5 μg/mL−15 μg/mL), as shown in Figure 3d. An interesting phenomenon was observed under the same concentration of the heavy metal solution; the removal rate of Cd^2+^ was faster than that of Cu^2+^ by TCPA. In addition, the removal percentage of Cu^2+^ or Cd^2+^ decreased with an increase in concentration. The reduced ionization produced on the TCPA surface and the hindrance of excess ions during adsorption contributed to this phenomenon [41].

#### 3.2.5. Adsorption Kinetics and Isotherms

In order to describe the adsorption process of Cu^2+^ or Cd^2+^ on adsorbents systematically and accurately, the data were fitted using a pseudo-first-order kinetic model (Equation (7)) and the pseudo-second-order kinetic model (Equation (8)).

(7) $$q_{t}=q_{e}\left(1-e^{-K_{1}t}\right)$$

(8) $$q_{t}=K_{2}q_{e}{}^{2}t/\left(1+K_{2}q_{e}t\right)$$

where *q_e_* and *q_t_* are the amounts of Cu^2+^ or Cd^2+^ adsorbed at equilibrium and at time t min, respectively, mg/g; *K*_1_ represents the rate constant of the pseudo-first-order kinetic model, min^−1^; *K*_2_ represents the rate constant of the pseudo-second-order kinetic model, g/(mg·min); t is adsorption time, min.

**Figure 4:**
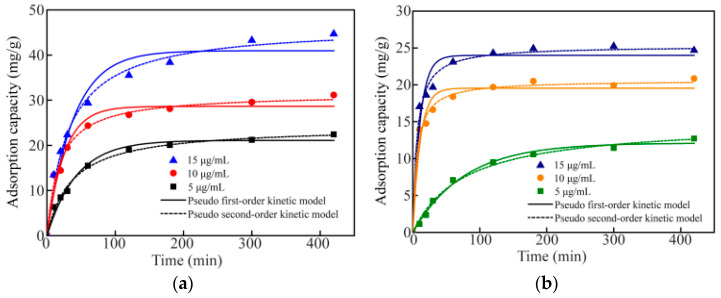

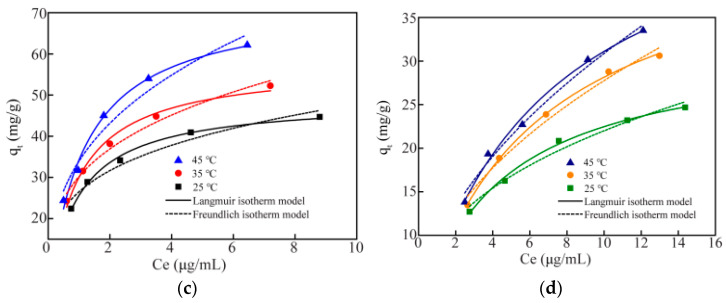
Data fit to the kinetic models for (**a**) Cu^2+^, (**b**) Cd^2+^ adsorption; data fit to the thermodynamic models for (**c**) Cu^2+^, (**d**) Cd^2+^ adsorption.

Figure 4 shows the adsorption kinetics and thermodynamics of Cu^2+^ or Cd^2+^ by TCPA. Figure 4a shows a high concentration of Cu^2+^ (15 μg/mL) in the solution; the maximum adsorption capacity that TCPA could reach would be 44.69 mg/g. The adsorption capacity of Cu^2+^ adsorbed by TCPA increased with the increase of the Cu^2+^ concentration in the solution. Meanwhile, the adsorption process of Cd^2+^ by TCPA showed similar results (Figure 4b). When the Cd^2+^ concentration was 15 μg/mL, the adsorption capacity of TCPA reached the maximum value (24.68 mg/g). At the same time, the adsorption capacity of Cu^2+^ (or Cd^2+^) increased with the increase in the concentration of Cu^2+^ (or Cd^2+^) in the solution, which was consistent with previous reports [42]. The high concentrations of Cu^2+^ or Cd^2+^ might provide a greater capture possibility for TCPA. In addition, the results for the adsorption process of Cu^2+^ (or Cd^2+^) by TCPA, which was fitted to the relevant adsorption data, are shown in Table 5. For the Cu^2+^ (or Cd^2+^) adsorption process by TCPA, the fitting results using the pseudo-second-order kinetic model were better than the pseudo-first-order kinetic model.

In order to describe the concentration relationship of Cu^2+^ (or Cd^2+^) in TCPA and solution at a certain temperature, the Langmuir isotherm model (Equation (9)) and Freundlich isotherm model (Equation (10)) were used to fit the relevant data.

(9) $$q_{e}=q_{m}K_{L}C_{e}/\left(1+bC_{e}\right)$$

(10) $$q_{e}=K_{F}C_{e}{}^{1/n}$$

where *q_e_* is the amounts of Cu^2+^ or Cd^2+^ in the TAAC at equilibrium; *q_m_* is the saturation capacity of the TCPA, mg/g; *C_e_* is the concentration of Cu^2+^ or Cd^2+^ at equilibrium, μg/mL; *K_L_* is the adsorption equilibrium constant of Langmuir isothermal model, L/mg; *K_F_* is the adsorption equilibrium constant of the Freundlich isothermal model, mg·g^−1^·(L·mg^−1^); 1/n is the adsorption intensity.

The adsorption isotherms of Cu^2+^ and Cd^2+^ by TCPA at different temperatures were investigated, as shown in Figure 4c,d. At different temperatures, the Cu^2+^+ or Cd^2+^ adsorption capacity of TCPA increased with the increase of equilibrium concentration. In addition, the adsorption capacity of Cu^2+^ (or Cd^2+^) adsorbed by TCPA increased with the increase of temperature, which indicated that the adsorption process was endothermic and increasing the temperature would facilitate the adsorption to proceed. The Langmuir and Freundlich isotherm models were employed to fit the relevant data, and the thermodynamic adsorption parameters are shown in Table 6. Compared with the Freundlich isotherm model, the Langmuir isotherm model was very close to the Cu^2+^ (or Cd^2+^) absorption at different temperatures, and there was a high correlation coefficient, indicating that the Cu^2+^ (or Cd^2+^) absorption was from a single layer [43]. At a temperature of 45 °C, the maximum saturation absorption of TCPA for Cu^2+^ and Cd^2+^ was 72.73 mg/g and 52.52 mg/g, respectively.

#### 3.2.6. Regeneration and Reusability of TCPA

**Figure 5:**
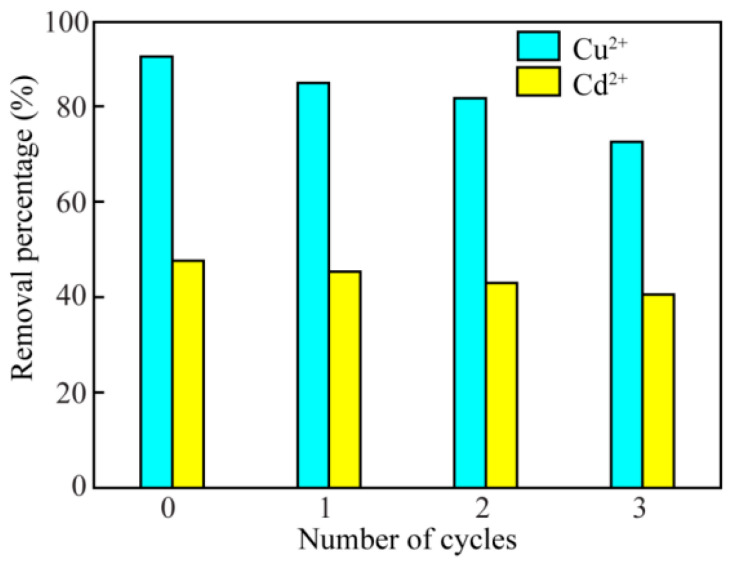
Removal percentage of TCPA by ternary cycles of regeneration at the Cu^2+^ or Cd^2+^ concentration of 5 μg/mL, pH = 6.0; adsorption time = 470 min, adsorption temperature = 25 °C.

In the process of actual application, the stability and regeneration of the adsorbent were important factors. TCPA had a high adsorption capacity at pH = 6.0. When the pH was low, it was beneficial to the desorption of Cu^2+^ (or Cd^2+^). Therefore, TCPA was desorbed by 0.1 mol/L HCl solution. As shown in Figure 5, the removal percentage of Cu^2+^ (or Cd^2+^) was not significantly reduced when TCPA was used for the third time. In general, TCPA had a large adsorption capacity for Cu^2+^ and Cd^2+^, indicating that TCPA has the potential for environmental treatment.

#### 3.2.7. Adsorption of Cu^2+^ and Cd^2+^ in a Binary Solution

Under natural conditions, a heavy metal rarely exists alone [44]. Moreover, different heavy metals would interact with each other in the same solution and affect the absorption of the adsorbent. Cu^2+^ and Cd^2+^ are common in industrial wastewater. Utilizing Cu^2+^ and Cd^2+^, the competitive adsorption characteristics in single and binary metal solutions were simulated; the results are shown in Table 7. In a low concentration of Cu^2+^ and Cd^2+^ binary solution, the adsorption capacity of Cu^2+^ or Cd^2+^ adsorbed by TCPA was similar to that in a single solution, which indicated that competition of active adsorptive sites was not frequent. However, with the increase of heavy metal (Cu^2+^ and Cd^2+^) concentration, the adsorption capacity of TCPA for Cu^2+^ and Cd^2+^ decreased slightly. These results clearly indicated that there was a competitive relationship between Cu^2+^+ and Cd^2+^ for the active adsorptive sites on the surface of TCPA at higher heavy metal concentrations.

#### 3.2.8. Comparison of Adsorption Performance of Different Biomass-Based Materials

TCPA had a three-dimensional porous structure and multi-functional surface adhesion layer of tannin and chitosan. TCPA also had a higher adsorption capacity for Cu^2+^ and Cd^2+^ than most bio-based adsorbents, as shown in Table 8. These results indicated that TCPA was a potential adsorbent for removing toxic metals from wastewater.

#### 3.2.9. Adsorption Mechanism of TCPA

**Figure 6:**
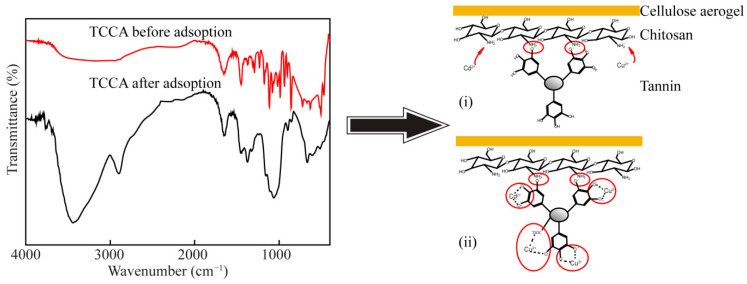
FTIR of before and after adsorption and possible mechanism of the adsorption of Cu^2+^ and Cd^2+^ on TCPA.

The process mechanisms of Cu^2+^ and Cd^2+^ adsorption could be deduced by comparing the FTIR of TCPA before and after heavy metals adsorption, as shown in Figure 6. The peaks of TCPA before adsorption at 3329 cm^−1^ (-OH), 1651 cm^−1^ (-NH_2_) and 1118 cm^−1^ (C-O-C) were replaced by the peaks of TCPA after adsorption at 3445 cm^−1^, 1450 cm^−1^ and 1045 ^−1^, respectively. Therefore, it was proved that the surface functional groups of TCPA interact with Cu^2+^ and Cd^2+^. Because bamboo pulp aerogels hardly adsorb Cu^2+^ and Cd^2+^, the mechanism of TCPA absorption mainly includes chitosan and tannin. (i) The amino group (-NH_2_) of the chitosan was a key functional group that could interact with Cd^2+^ and Cu^2+^ [48]. Furthermore, amines (-NH_2_) were positively charged in slightly acidic environments and existed in the form of (NH_3_^+^), thus providing more binding points for tannin. After TCPA adsorbed heavy metals, the peak of 1650 cm^−1^ disappeared, indicating that Cu^2+^ and Cd^2+^ were adsorbed on the amino sites of chitosan by electrostatic interaction. (ii) The tannin in TCPA reacted with Cu^2+^ and Cd^2+^ at the adsorption of heavy metals mainly by phenolic hydroxyl groups, and the other reaction was to combine Cu^2+^ and Cd^2+^ through ion exchange on carboxyl and hydroxyl groups [49].

## 4. Conclusions

In summary, an environmentally friendly, low-cost, renewable and low-density TCPA adsorbent was successfully synthesized through a simple freeze-drying process and analyzed by FTIR, XPS, SEM, TEM, TGA and surface area and porosity methods. TPCA exhibited 3D porous morphology with a large number of micropores and was mesoporous, had a large specific surface area (137.33 m^2^/g) and low density (48.44 mg/g). It had a large number of amino, carboxyl and hydroxyl groups on its surface due to the modification of chitosan and tannic acid, which played a key role in the efficient and synergistic removal of Cu^2+^ and Cd^2+^. The maximum adsorption capacity for Cu^2+^ and Cd^2+^ of the TCPA was 72.73 mg/g and 52.52 mg/g, respectively, which was higher than most of the reported biological-based adsorbents. The adsorption process for Cu^2+^ and Cd^2+^ by TCPA followed the pseudo-second-order model and Langmuir isotherm mode, and it was a spontaneous endothermic adsorption process. TCPA could be regenerated and reused efficiently three times by using an HCl solution as the eluent. This research showed a green and simple strategy using bamboo pulp, a modified natural polysaccharide, and polyphenol. It broadens the field of application for bamboo pulp and has potential value in the purification of heavy metal wastewater.

## Figures and Tables

**Table 1:** The reagent dosage in the fabrication procedure.

Sample	Bamboo Pulp (g)	Tannin (g)	Chitosan (g)
PA	0.5	/	/
TPA	0.5	0.04	/
CPA	0.5	/	0.04
TCPA	0.5	0.02	0.02

**Table 2:** Surface element composition of PA, TPA, CPA and TCPA.

Sample	C (%)	O (%)	N (%)	O/C Ratio
PA	62.55	37.45	/	0.60
CPA	62.15	32.92	4.93	0.53
TCPA	70.38	27.48	2.14	0.39

**Table 3:** Physical properties of as-synthesized samples.

Sample	S_BET_ (m^2^/g)	D_pore_ (nm)	V_totalpore_ (cm^3^/g)	V_<50 nm_ (cm^3^/g)	V_<50 nm_/V_totalpore_	ρ (mg/cm^3^)
PA	112.60	9.43	0.263	0.202	0.768	46.29
TCPA	137.33	9.36	0.315	0.261	0.829	48.44

**Table 4:** Thermodynamic parameters of Cu^2+^ or Cd^2+^ adsorption on the TCPA.

Adsorbate	Δ*G^θ^* (kJ/mol)			Δ*H^θ^* (kJ/mol)	Δ*S^θ^* (kJ/mol·K)
	25 °C	35 °C	45 °C		
Cu^2+^	−6.91	−7.61	−8.12	13.95	0.070
Cd^2+^	−2.67	−3.29	−3.77	15.82	0.062

**Table 5:** Kinetic model parameters for Cu^2+^ or Cd^2+^ adsorption on the TCPA.

Adsorbate	Concentration (mg/L)	Pseudo 1st Order Kinetic			Pseudo 2nd Order Kinetic		
		*K*_1_ (min^−1^)	*q_e_* (mg/g)	R^2^	*K*_2_ (g/mg·min)	*q_e_* (mg/g)	R^2^
Cu^2+^	5	0.023	21.12	0.982	0.0012	24.17	0.993
	10	0.040	28.65	0.957	0.0017	31.43	0.985
	15	0.026	40.97	0.957	0.0007	46.56	0.991
Cd^2+^	5	0.013	12.12	0.991	0.0009	14.94	0.992
	10	0.092	19.55	0.951	0.0080	20.62	0.989
	15	0.092	23.99	0.953	0.0066	25.28	0.989

**Table 6:** Adsorption isothermal model parameters for Cu^2+^ or Cd^2+^ adsorption.

Adsorbate	Temperature (°C)	Langmuir Isotherm			Freundlich Isotherm		
		*q_m_* (mg/g)	*K_L_*	R^2^	n	*K_F_*	R^2^
Cu^2+^	25	48.91	1.10	0.994	3.91	26.50	0.950
	35	57.51	1.09	0.983	3.45	30.20	0.977
	45	72.73	0.89	0.988	2.89	33.95	0.950
Cd^2+^	25	32.15	0.23	0.995	2.57	9.00	0.972
	35	45.77	0.16	0.998	2.04	9.00	0.979
	45	52.52	0.15	0.992	1.90	9.18	0.985

**Table 7:** Adsorption of Cu^2+^ and Cd^2+^ from single and binary adsorbates by the TCPA.

Concentration (μg/mL)	Adsorption Capacity (mg/g)		
	Cu^2+^	Cd^2+^	Cu^2+^ + Cd^2+^
5 μg/mL Cu^2+^ or 5 μg/mL Cd^2+^	22.41	12.71	/
5 μg/mL Cu^2+^ + 5 μg/mL Cd^2+^	20.89	13.45	34.34
10 μg/mL Cu^2+^ or 10 μg/mL Cd^2+^	34.14	20.85	/
10 μg/mL Cu^2+^ + 10 μg/mL Cd^2+^	31.57	17.36	48.93
15 μg/mL Cu^2+^ or 15 μg/mL Cd^2+^	44.69	24.68	/
15 μg/mL Cu^2+^ + 15 μg/mL Cd^2+^	39.28	22.52	61.80

Note: adsorption temperature = 25 °C, adsorption time = 470 min, pH = 6.0.

**Table 8:** Comparison of TCPA with other biomass-based materials.

Adsorbent	Initial Concentration (μg/mL)		Adsorption Capacity (mg/g)		Ref.
	Cu^2+^	Cd^2+^	Cu^2+^	Cd^2+^	
Polyacrylic acid grafted with lignin	63.55	112.41	20.69	6.608	[45]
Wood ash amended biochar	10	10	38.90	10.02	[46]
*Moringa oleifera* seed	20	20	3.64	5.03	[47]
Functional paper material	200	200	5.07	6.02	[43] Converted by 135 g/m^2^
TCPA	5	5	72.73	52.52	This work

## Data Availability

The data presented in this study are available on request from the corresponding author.
